# Effects of sotagliflozin on kidney and cardiac outcome in a hypertensive model of subtotal nephrectomy in male mice

**DOI:** 10.14814/phy2.70217

**Published:** 2025-03-28

**Authors:** Helen Goodluck, Alice Zemljic‐Harpf, Ony Araujo Galdino, Sadhana Kanoo, Natalia Lopez, Young Chul Kim, Volker Vallon

**Affiliations:** ^1^ Division of Nephrology & Hypertension, Department of Medicine University of California San Diego, and VA San Diego Healthcare System San Diego California USA; ^2^ Department of Clinical and Toxicological Analyses Federal University of Rio Grande do Norte (UFRN) Natal RN Brazil

**Keywords:** cardiac hypertrophy, GFR, kidney injury, SGLT1 inhibition, SGLT2 inhibition

## Abstract

Dual inhibition of sodium glucose cotransporters 1 and 2 (SGLT1/SGLT2) by sotagliflozin protects the kidney and heart in patients with type 2 diabetes mellitus (T2DM) and chronic kidney disease (CKD). To gain mechanistic insights, the current study aimed to establish a murine model of hypertensive CKD that shows cardio‐renal protection by sotagliflozin. Since protection by SGLT2 inhibitors can be diabetes‐independent, a nondiabetic murine model of subtotal nephrectomy with angiotensin II infusion‐facilitated hypertension was followed for 7 weeks. The model showed 40% lower GFR, doubling in plasma FGF23, 50 mmHg higher systolic blood pressure (SBP), 100‐fold increased albuminuria, and robust signs of kidney injury, inflammation, and fibrosis versus sham controls, associated with a 30% larger left cardiac ventricle and wall thickness and upregulation of markers of cardiac overload and fibrosis. Sotagliflozin, initiated 1 week after the last surgery, showed target‐engagement evidenced by glucosuria, 9 mmHg lower SBP, temporal reduction in body weight and GFR, and 30% higher plasma GLP1. Sotagliflozin, however, did not improve markers of kidney injury, inflammation, fibrosis, albuminuria, and plasma FGF23, or signs of cardiac overload, fibrosis, or impaired function. Limited sotagliflozin responsiveness may relate to short treatment time, limited metabolic benefits in nondiabetic setting and/or the model's dominant angiotensin II‐driven effects/hypertension.

## 1 INTRODUCTION

Patients with chronic kidney disease (CKD) carry a high risk of cardiovascular complications (Gansevoort et al., 2013). Kidney risk reduction has been established for angiotensin converting enzyme (ACE) inhibitors or angiotensin receptor blockers (ARBs), and more recently, for inhibitors of the sodium‐glucose cotransporter 2 (SGLT2i), in both cases independent of sex (American Diabetes Association Professional Practice Committee, 2024; Rossing et al., 2022). The primary effect of SGLT2i on the kidney is the inhibition of glucose transport in the early proximal tubule. The resulting enhanced glucose load to downstream tubular segments is partially compensated by the sodium‐glucose cotransporter, SGLT1. The remaining glucose is lost into the urine, accompanied by an osmotic diuresis. Clinical outcome trials in individuals with and without type 2 diabetes (T2DM) have established that SGLT2i can protect the kidneys and heart from failing, but the involved mechanisms remain incompletely understood (Vallon, 2024).

Mediation analyses linked SGLT2i‐induced lowering of plasma volume or increase in hematocrit, and the reduction in serum urate levels and albuminuria to kidney and heart protection (Fitchett et al., 2021; Inzucchi et al., 2018; Li, Neal, et al., 2020; Li, Woodward, et al., 2020). These clinical phenotypes can be explained by the effects of SGLT2 inhibition on the early proximal tubule (Vallon, 2024). Briefly, the physiology of tubule‐glomerular feedback explains the acute lowering of GFR and glomerular capillary pressure in response to SGLT2i, which reduces albuminuria but also contributes to long‐term GFR preservation, in part by lowering the filtered tubular load and thereby oxygen demand in kidney cortex (Vallon & Thomson, 2020). In the early proximal tubule, SGLT2 is functionally coupled to other sodium and metabolite transporters, and, as a consequence, SGLT2i initially excrete more sodium than expected and are uricosuric (Billing et al., 2024; Novikov et al., 2019; Onishi et al., 2020; Rao et al., 2024; Suijk et al., 2022), which lowers plasma volume, blood pressure, and serum levels of urate. SGLT2i lower gluco‐toxicity in early proximal tubules, and the downstream transport shift may simulate “systemic hypoxia” and trigger erythropoietin release, which, together with the osmotic diuresis, increases hematocrit and thereby blood oxygen delivery (Layton & Vallon, 2018). Last but not least, the glucosuria‐induced insulin‐sparing and fasting‐like metabolic phenotype of SGLT2i can be organ‐protective (Vallon, 2024).

Nevertheless, there is a need for additional and complementing strategies since SGLT2i can attenuate but not halt the progressive loss of kidney function (Heerspink et al., 2021; Perkovic et al., 2019) and the effects of SGLT2i on cardiovascular outcomes like stroke and myocardial infarction are limited (Pitt et al., 2022). As mentioned above, SGLT1 is expressed in the late proximal tubule and thick ascending limb where it can reabsorb about 40% of the filtered glucose, when upstream SGLT2 is inhibited under euglycemic conditions (Rieg et al., 2014). As a consequence, dual inhibition of SGLT2 and SGLT1 induces a stronger glucosuric and blood glucose lowering effect (Powell, DaCosta, et al., 2013; Rieg et al., 2014; Song et al., 2019). Moreover, SGLT1 expression is not restricted to the kidney but SGLT1 can be detected in many organs including the small intestine and the heart (Song et al., 2016). Inhibition of intestinal SGLT1 has a dual effect on glucose homeostasis, (i) a direct effect by inhibiting glucose uptake in the small intestine and thereby delaying postprandial glucose absorption (Gorboulev et al., 2012; Turk et al., 1991) and (ii) an indirect effect via effects on the microbiome and promoting a sustained release of glucose‐lowering incretin hormones like GLP‐1 (Dobbins et al., 2015; Goodwin et al., 2017; Powell, Smith, et al., 2013). SGLT1 has also been localized to the brain and heart, including in capillaries, and its inhibition has been linked to improved outcome in stroke and myocardial infarction (Pitt et al., 2022). How SGLT1i reduces stroke and myocardial infarction is an active field of research, but may relate to the finding that an increase in GLP‐1 can decrease platelet activation and enhance atherosclerotic plaque stability (Cefalo et al., 2019; Pitt et al., 2022).

Sotagliflozin is a dual SGLT1 and 2 inhibitor, and from the currently available evidence (SCORED and SOLOIST trial), the compound is likely at least as effective as the more selective SGLT2is in preventing heart failure (HF) in patients with T2DM and CKD as well as in reducing cardiovascular mortality and hospitalization for HF in patients with T2DM and HF with reduced or preserved ejection fraction (HFrEF, HFpEF) (Bhatt, Szarek, Pitt, et al., 2021; Bhatt, Szarek, Steg, et al., 2021). The SGLT1 inhibition component appears to add to the benefits of SGLT2i in that sotagliflozin reduced nonfatal and fatal stroke as well as nonfatal and fatal MI in patients with T2DM by >30% (Bhatt, Szarek, Pitt, et al., 2021; Bhatt, Szarek, Steg, et al., 2021). As a consequence, sotagliflozin has been approved by the Food and Drug Administration to reduce the risk of cardiovascular death, hospitalization for HF, and urgent HF visits in adults with HF or T2DM, CKD, and other CV risk factors.

Beyond the cardioprotective benefits, sotagliflozin significantly reduced albuminuria and the risk of progression of albuminuria in patients with T2DM and CKD (Cherney et al., 2023). Moreover, the incidence of the composite kidney end point of a sustained ≥50% decrease in eGFR, sustained eGFR of <15 mL/min per 1.73 m^2^, dialysis, or kidney transplant was reduced by 29% in the SCORED trial, albeit this did not reach statistical significance (Bhatt, Szarek, Pitt, et al., 2021). The SCORED trial was stopped early because of lack of funding, which limited the median duration of follow‐up to only 16 months and the number of events and statistical power to assess some outcomes, such as CKD progression. In an exploratory analysis of the SCORED trial, and by using the complete laboratory dataset, sotagliflozin was found to reduce the risk of kidney and cardiorenal composite endpoints versus placebo in patients with T2DM and CKD (Sridhar et al., 2024).

There is a need to evaluate sotagliflozin in the nondiabetic setting and gain a deeper mechanistic understanding. Therefore, the goal of the current study was to establish and explore a nondiabetic mouse model of hypertensive CKD in which sotagliflozin has kidney and heart protective effects. The model could then serve for follow up studies to gain more mechanistic insights (e.g., by the use of mice lacking Sglt1). C57BL/6 mice, which are often used for gene‐targeted mouse models, show little if any signs of hypertension and CKD in response to kidney mass resection (Leelahavanichkul et al., 2010). Co‐infusing C57BL/6 mice with low‐dose angiotensin II (AngII) restores the blood pressure increase after subtotal nephrectomy (STN) observed in other mouse strains and makes the C57BL/6 mouse strain susceptible to CKD (Leelahavanichkul et al., 2010). Thus, the current study explored the effects of sotagliflozin in the described hypertensive murine STN model that is characterized by robustly reduced GFR and increased albuminuria, compensatory kidney growth and signs of kidney injury, inflammation, and fibrosis as well as cardiac hypertrophy and fibrosis.

## 2 MATERIALS AND METHODS

### 2.1 Animals

**FIGURE 1:**
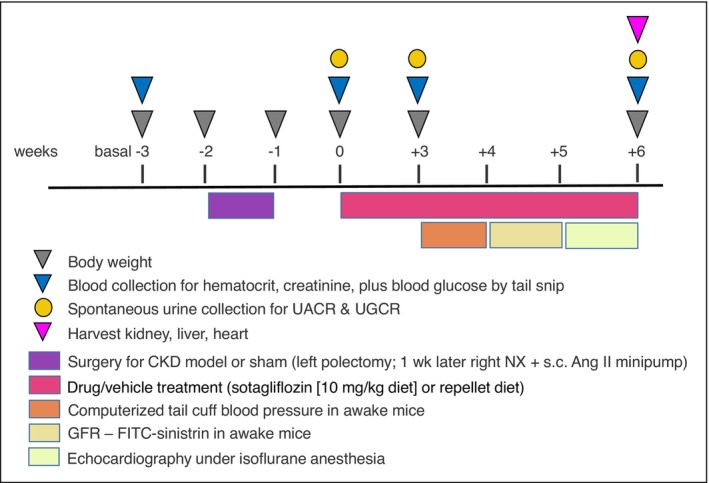
Experimental Plan. Experiments were conducted on adult male C57BL/6J mice. Baseline measurements (“Basal”) were taken before surgery. One week later (−2 weeks), the hypertensive subtotal nephrectomy (STN) model was initiated by a two‐step subtotal nephrectomy combined with continuous angiotensin II infusion through a s.c. minipump. One‐week postsurgery (0 week), measurements were repeated, and treatment with sotagliflozin or vehicle was initiated. After 3 weeks of treatment (+3 weeks), additional measurements were taken and blood pressure measured in the following week. Glomerular filtration rate (GFR) was measured in the 5th week of treatment, and echocardiography in the 6th week. At the end of the 6th week of treatment, kidneys and hearts were harvested for further analysis; UACR, urinary albumin to creatinine ratio; UGCR, urinary glucose to creatinine ratio.

All animal experimentation was conducted in accordance with the Guide for Care and Use of Laboratory Animals (National Institutes of Health, Bethesda, MD) and was approved by the local Institutional Animal Care and Use Committee. Studies in humans indicate that cardiorenal protection by sotagliflozin is independent of sex (Bhatt, Szarek, Pitt, et al., 2021; Bhatt, Szarek, Steg, et al., 2021; Sridhar et al., 2024). Therefore, the current study focused on one sex. Eight‐week‐old male C57BL/6 mice (Stock No: 000664) were purchased from Jackson laboratories. All animals were housed in the same animal room with a 12:12 h light–dark cycle and free access to standard chow (Envigo, Cat# 7001). Figure 1 provides an overview of the experimental plan.

### 2.2 Hypertensive subtotal nephrectomy (STN) model

At the age of 10–12 weeks, STN was created by two‐step subtotal nephrectomy, and combined with continuous subcutaneous angiotensin II (AngII) infusion. The approach is adapted from the original description by Leelahavanichkul et al. who showed that infusion of AngII elevates blood pressure and enhances kidney susceptibility of C57BL/6 mice to the subtotal nephrectomy model. Briefly, one week after polectomy of the left kidney, the right kidney was removed and an osmotic minipump implanted subcutaneously into the right flank to deliver AngII (see details below). The weight of removed kidney tissue was determined. Both surgeries were performed under ketamine (100 mg/kg body wt i.p., Henry Schein Animal Health, Dublin, OH) and xylazine (10 mg/kg body wt i.p.; Bimeda‐MTC Animal Health, Cambridge, ON, Canada) anesthesia and body temperature control (36–37°C, using a heated surgical table and rectal temperature probe). Buprenorphine was applied (50 μg/kg s.c.; provided by the VA San Diego Hospital Pharmacy) for postsurgery pain management, and warm sterile saline (0.9% NaCl) was injected (30 μL/g body wt s.c.) to replenish lost body fluid. Mice were put back in clean home cages (on heating pad) and allowed to recover (~2 h). Upright and mobile animals were given a dose of slow‐release buprenorphine (50 μg/kg s.c.) and provided with diet gels (ClearH2O Inc., Westbrook, ME) and a bottle of water for facilitated access to food and fluid. Additional buprenorphine was administered s.c. 6–12 h after surgery as needed for up to 3 days post‐op. Sham mice received the same two episodes of anesthesia and surgery and kidney manipulations except that no polectomy or heminephrectomy was performed and no minipump implanted.

AngII (Cat# 002–12, Phoenix Pharmaceuticals, Inc., USA) was formulated in 0.9% normal sterile saline with 5% acetic acid to deliver a dose of 0.75 μg/kg/min over a 42‐day period. 250 μL of the AngII solution was added per minipump (Alzet model 2006 infusion pump; DURECT Corporation USA, ALZET Osmotic Pumps). Pumps were kept in 37° sterile saline for 12–24 h prior to implantation to prime the pump. After priming, the full infusion rate (0.15 μL/hr) is reached after 40 h according to the manufacturer.

One week after the final surgery, the diet for half of the nephrectomized mice was switched to the standard chow containing sotagliflozin (10 mg/kg diet; compound provided by Lexicon Pharmaceuticals) based on a targeted dose of sotagliflozin of 10 mg/kg body weight and day. The other half of nephrectomized mice and the sham controls were switched to repelleted control diet. Treatments were not blinded.

### 2.3 Sample collection and measurement of blood pressure and GFR in awake mice

One week before the surgery (basal: time − 3 weeks) as well as before the start of treatment (time 0) and after week 3 of treatment, body weight was determined in nonfasted and conscious mice between 9 and 10 am, followed by retro‐orbital blood collection under isoflurane anesthesia to determine hematocrit and plasma creatinine (see details below).

In the 4th week of treatment, systolic blood pressure and heart rate were determined in awake mice by an automated tail cuff system (BP‐2000 Blood Pressure Analysis System™, Visitech‐Systems, Apex, NC) for six consecutive days after appropriate training (Onishi et al., 2020; Song et al., 2019). The automated tail cuff blood pressure approach is useful to measure blood pressure when certain precautions to reduce the stress of the animals are considered (Krege et al., 1995; Meneton et al., 2000), including appropriate training of the mice over multiple days, prewarming to an ambient temperature of 29°C, measurement in a quiet and semi‐darkened and clean environment, and performance of the measurements by one person and during a defined daytime, when blood pressure is stable (between 1:00 and 3:00 pm). All these precautions were taken in the present study.

In the 5th week of treatment, GFR was determined (between 9 am to 12 noon) using plasma elimination kinetics of FITC‐sinistrin (Cat# FTC‐FS001, Fresenius‐Kabi, Linz, Austria) measured by a transdermal detection system (NIC‐Kidney Device, Medibeacon) (Scarfe et al., 2018). Briefly, under short and mild isoflurane anesthesia, a bolus dose of FITC‐sinistrin (4%, 2 μL/g body weight in 0.85% NaCl) was injected into the retro‐orbital plexus, followed by quick recovery of the mice from anesthesia. The transdermal signal was monitored for 2–3 min before FITC‐sinistrin injection for baseline measurements and over 1.5 hrs after injection. Subsequently and after device readout (by MBLAB software, MPD Lab Version 2.2), the data were analyzed and GFR calculated using manufacturer software (MB Studio2).

### 2.4 Transthoracic echocardiography

In the 6th week of treatment, echocardiography (ECG, M‐mode, two‐dimensional tissue Doppler, and pulse‐wave Doppler) was performed under isoflurane anesthesia (1%–1.5% at a flow rate of 1 L/min oxygen) using a small‐animal, high‐resolution Vevo 3100 imaging unit with a MX550S transducer (~26–52 MHz) transducer and VevoLab 3.2.0 software (VisualSonics Inc., Toronto, Canada, Sonosite, Fujifilm). To avoid anesthesia induced changes in cardiac contractility and relaxation (Okada et al., 2011; Rottman et al., 2007), heart rates were maintained between 550 and 600 bpm for the assessment of systolic function (M‐Mode) and Tissue Doppler analysis. Left‐ventricular ejection fraction (%EF), fractional shortening (%FS), and wall and wall thicknesses were assessed as previously described (Nogueira et al., 2022; Zemljic‐Harpf et al., 2007). Tissue Doppler imaging was also used to measure early diastolic mitral annular velocity (E′). This parameter evaluates left ventricular myocardial relaxation in the longitudinal direction (Okada et al., 2011). E is the early diastolic transmitral valve flow velocity measured by pulse‐wave Doppler. The ratio of these values (E/E′) estimates left ventricle filling pressure. Late diastolic mitral annular velocity (A) was also measured by pulse‐wave Doppler. Thus, E'/A' is used to assess diastolic function by early (E) to late (A) diastolic transmitral valve blood flow velocities (Nogueira et al., 2022; Zemljic‐Harpf et al., 2021). Heart rates were maintained between 350 and 450 bpm for pulse wave Doppler analysis to measure mitral inflow representative E‐ and A‐waves (to ensure E‐ and A‐wave separation in mice). The sonographer was blinded to the experimental groups. Fractional shortening (FS) and Ejection fraction (EF) were determined using formulas as described:

$$\begin{array}{c} \%\mathrm{FS}=\left[\frac{\text{LVIDd}-\text{LVIDs}}{\text{LVIDd}}\right]\times 100\;\%\mathrm{EF}=\left[\frac{L{\text{VIDd}}^{3}-{\text{LVIDs}}^{3}}{{\text{LVIDd}}^{3}}\right]\times 100, \end{array}$$

where LVIDd and LVIDs are left ventricular internal diameters at end diastole and end systole, respectively.

At the end of the 6th week of treatment, blood glucose was determined between 9 and 10 am in awake mice by tail snip followed by spot urine collection and measuring body weight. Mice were then anesthetized with intraperitoneal injection of a ketamine (Henry Schein Animal Health, Dublin, OH, USA) and xylazine (Bimeda‐MTC Animal Health, Cambridge, ON, Canada) cocktail (100 mg/kg and 10 mg/kg, respectively) followed by retro‐orbital blood collection and subsequent harvesting of kidneys, liver and heart. Kidneys were gently decapsulated, and organ wet weights quickly determined. Organs were snap frozen in liquid N_2_ and stored at −80°C for quantitative PCR analysis.

### 2.5 Blood and urine analysis

Blood glucose levels were determined using the Ascensia Elite XL glucometer (Bayer, Mishawaka, IN). Urine albumin was determined using a commercial assay (Cat# E99‐1324, FORTIS Life Sciences, Boston, MA) (Oe et al., 2024). Concentrations in spot urine were normalized to creatinine concentration, which was measured by a kinetic modification of the Jaffe's reaction (Thermo Fisher Scientific, Waltham, MA) (Oe et al., 2024).

### 2.6 Quantitative reverse transcription polymerase chain reaction (qRT‐PCR)

RNA from kidney cortex was isolated using a mechanical tissue homogenizer in lysis buffer by RNeasy Plus Mini Kit (Cat# 74134, Qiagen, Germantown, MD) and cDNA was prepared using the SuperScript IV First‐Strand Synthesis System (Cat# 18090200, Thermo Scientific, Louisville, CO) as per the provided manufacturer's instruction (Oe et al., 2024). TaqMan Universal PCR Master Mix (Cat# 4304437, Applied Biosystems) and specific primers (listed in Table 1, ThermoFisher Scientific) were used in a 7500 Real‐Time PCR System (2 min at 50°C, 10 min at 95°C with 40 cycles of 15 s at 95°C and 1 min at 60°C) for quantification purposes. Expression levels were expressed as relative fold increases/decreases normalized to housekeeping genes rpl19 and hprt, which were not different between groups (not shown). Each experiment was performed in duplicate.

**TABLE 1:** Real‐time PCR primers used.

Target gene	Assay ID
*Acta1*	Mm00808218_g1
*Ccl2* (MCP1)	Mm00441242_m1
*Ccr2*	Mm04207877_m1
*Col1a1*	Mm00801666_m1
*Fn1* (fibronectin)	Mm01256744_m1
*Harvc1* (KIM1)	Mm00506686_m1
*Hprt*	Mm00446968_m1
*Il‐6*	Mm00446190_m1
*Lcn2* (NGAL)	Mm01324470_m1
*Nppa*	Mm01255747_g1
*Nppb*	Mm01255770_g1
*Mmp2*	Mm00439498_m1
*renin*	Mm02342889_g1
*Rpl19*	Mm02601633_g1
*Slc5a1* (SGLT1)	Mm00451203_m1
*Slc5a2* (SGLT2)	Mm00453831_m1
*Tgfb1*	Mm01178820_m1
*Timp1*	Mm01341361_m1
*Tnfa*	Mm00443258_m1

*Note*: The TaqMan Assays were purchased from ThermoFisher Scientific.

Abbreviations: Ccl2 (MCP1), monocyte chemoattractant protein‐1/chemokine (C‐C motif) ligand 2; Ccr2, C‐C motif chemokine receptor 2; Col1a1, collagen type I‐α1; Havcr1 (KIM‐1), kidney injury molecule‐1/hepatitis A virus cellular receptor 1; Hprt, hypoxanthine phosphoribosyltransferase 1; Il‐6, interleukin‐6; Lcn2/NGAL, human neutrophil gelatinase‐associated lipocalin; Rpl19, ribosomal protein L19; TGFβ1, transforming growth factor beta‐1; TIMP1, tissue inhibitor of metalloproteinases 1; TNFα, tumor necrosis factor alpha.

### 2.7 Histological fibrosis analysis

Formalin‐fixed paraffin‐embedded kidney and cardiac samples (5 μm) were prepared for histological analyses by the University of California‐San Diego Tissue Technology Shared Resource (supported by National Cancer Institute Cancer Center Support Grant P30‐CA‐23100). Sirius Red (Cat# NC9039835, Fisher Scientific) staining was performed to evaluate kidney fibrosis area in scanned images as described (Vallon et al., 2013). To this end, the ratio of red color area to total renal cortex and medulla area was measured using ImageJ software (v.1.53, NIH; https://imagej.nih.gov/ij). Cardiac fibrosis was analyzed using Masson's Trichrome and ImageJ software analysis (Vallon et al., 2006). For both analyses, the investigator was blinded to study groups.

### 2.8 Statistical analysis

Values that differed by more than two standard deviations from the group mean were considered as outliers and removed from the analysis. To analyze for statistical differences between the three groups, one‐way ANOVA and Holm‐Sidak post hoc versus STN‐AngII vehicle was performed for normally distributed data; Kruskal–Wallis test by ranks followed by Dunn post hoc versus STN‐AngII veh was performed for non‐normally distributed data. *p* < 0.05 was considered statistically significant.

## 3 RESULTS

### 3.1 Basal measurements before surgery, establishing subtotal nephrectomy (STN)‐angiotensin II (AngII) model, and measurements before first treatment

Before surgery, mice were randomly assigned to the three groups, while confirming that basal measurements of body weight, hematocrit and plasma creatinine were similar among groups (Figure S1). To induce the STN model, similar masses of kidney tissue (left kidney polectomy and right nephrectomy) were resected in both groups (Figure S1B). Combined with s.c. implantation of AngII‐delivering minipumps, this resulted in similar increases in plasma creatinine and urine albumin to creatinine ratios (UACR) in both STN‐AngII groups versus sham operated mice (Figure S1C). The STN‐AngII model reduced urine creatinine concentrations, indicating dilute urine, while body weight, hematocrit and urine glucose to creatinine ratios (UGCR) were not different among the three groups before the first drug application (Figure S1C).

### 3.2 Phenotype of vehicle‐treated STN‐AngII model

**FIGURE 2:**
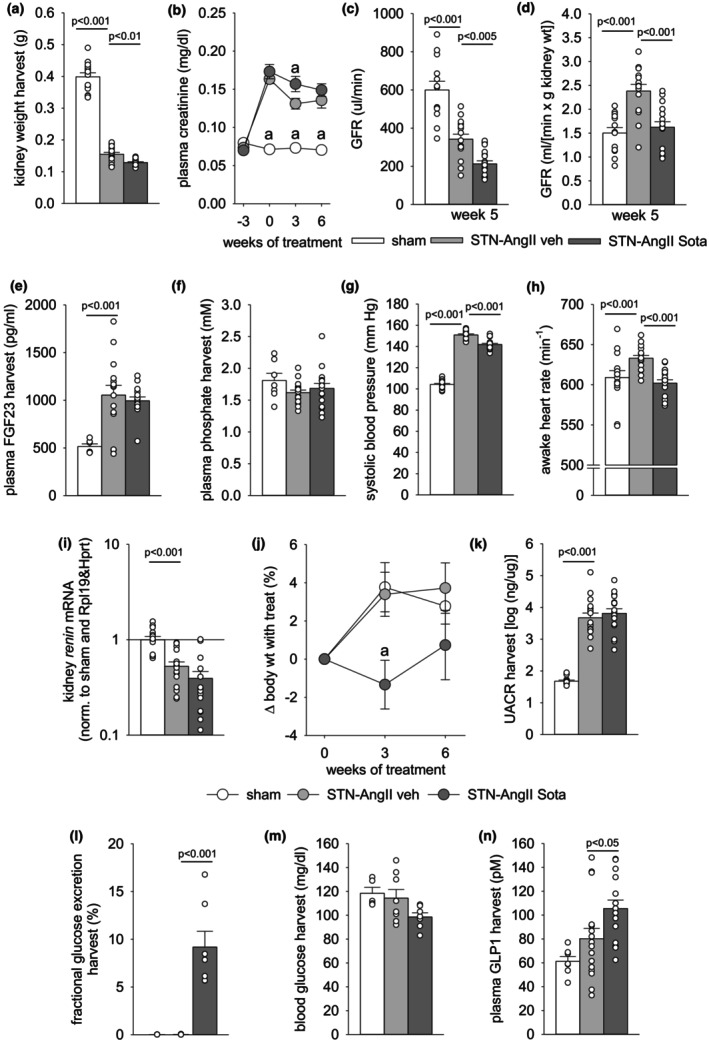
Impact of STN‐AngII model and sotagliflozin on kidney weight, markers of kidney function, blood pressure, albuminuria, glucosuria and plasma glucagon‐like peptide‐1 (GLP‐1) levels. (a) Kidney weight at harvest; (b) Plasma creatinine profile; (c) Glomerular filtration rate (GFR) at week 5; (d) GFR per gram of kidney weight at week 5; (e) Plasma fibroblast growth factor 23 at harvest; (f) Plasma phosphate at harvest; (g) Systolic blood pressure; (h) Heart rate; (i) Kidney renin mRNA expression (normalized to sham and Rpl19 & Hprt); (j) Percent change in bodyweight in response to treatment; (k) Urinary albumin‐to‐creatinine ratio at harvest; (l) Fractional glucose excretion; (m) Blood glucose; (n) Plasma glucagon‐like peptide‐1 (GLP1) levels at harvest. Individual values and/or means±SE are shown. For normally distributed data, one‐way ANOVA and Holm‐Sidak post hoc vs. STN‐AngII veh; for non‐normally distributed data, one‐way ANOVA on ranks and Dunn post hoc vs. STN‐AngII veh; *n* = 13–19/group for all parameters except *n* = 6–17/group for E, F, N and *n* = 5–8/group for L and M. a, *p* < 0.05 versus STN‐AngII veh.

**FIGURE 3:**
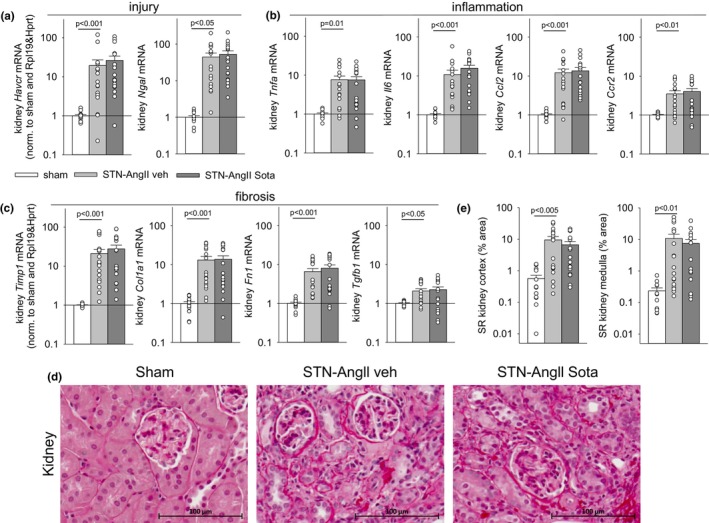
Sotagliflozin did not affect makers of kidney injury, inflammation or fibrosis in the STN‐AngII model. Kidney mRNA expression (normalized to sham and housekeeping genes Rpl19 & Hprt) of markers of (a) Tubular injury [hepatitis A virus cellular receptor 1 (HAVCR1), neutrophil gelatinase‐associated lipocalin (NGAL)]; (b) Inflammation [tumor necrosis factor‐alpha (TNF‐α), interleukin‐6 (IL‐6), C‐C motif chemokine ligand 2 (CCL2), C‐C motif chemokine receptor 2 (CCR2)]; (c) Fibrosis [TIMP metallopeptidase inhibitor 1 (TIMP‐1), collagen type I alpha 1 (COL1A1), fibronectin 1 (FN1), transforming growth factor beta‐1 (TGF‐β1)]; (d) Representative images of Picrosirius Red‐stained cortical tissue sections for fibrosis staining; (e) Semiquantitative analysis of the percentage area of fibrosis in kidney cortex and medulla. Individual values and/or means ± SE are shown. For normally distributed data, one‐way ANOVA and Holm‐Sidak post hoc versus STN‐AngII veh; for non‐normally distributed data, one‐way ANOVA on ranks and Dunn post hoc versus STN‐AngII veh; *n* = 13–19/group for all parameters.

At harvest, the vehicle‐treated STN‐AngII group had a ~60% lower kidney weight versus the sham group (Figure 2a). This was associated with elevated plasma creatinine levels (Figure 2b) and a 40% lower GFR when measured in the 5th week of vehicle treatment (Figure 2c and Figure S2A). Considering approximately resection of ~5/6 of nephrons, the total GFR values suggested relative hyperfiltration in remnant nephrons. Plasma FGF23 was doubled to stabilize plasma phosphate levels (Figure 2e,f). The STN‐AngII model induced a robust, ~50 mmHg increase in systolic blood pressure (SBP, Figure 2g) associated with a small increase in heart rate (Figure 2h). The rise in SBP together with a modest suppression of kidney renin mRNA expression (Figure 2i) seemed effective in stabilizing body fluid balance as indicated by maintained body weight versus sham (Figure 2j) while maintaining normal food intake (Figure S2B). The elevated blood pressure and the hyperfiltration in remaining nephrons facilitated a ~100‐fold increase in UACR (Figure 2k), associated with robust gene expression upregulation of kidney markers of injury (Kim1, Ngal—Figure 3a), inflammation (Tnfa, Il6, Ccl2, Ccr2—Figure 3b), and fibrosis (Timp1, Col1a1, Fn1, Tgfb1—Figure 3c). The latter was confirmed by enhanced Sirius red (SR) fibrosis staining in kidney cortex and medulla (Figure 3d,e).

**FIGURE 4:**
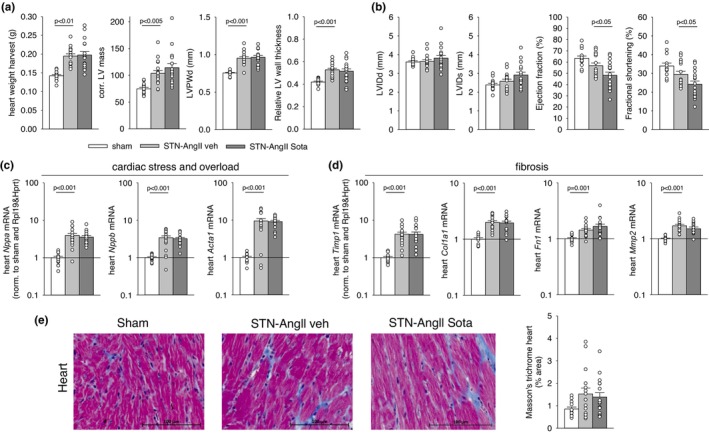
Sotagliflozin did not affect cardiac hypertrophy or markers of cardiac stress and fibrosis while modestly reducing heart contractility in the STN‐AngII model. (a) Measures of heart size and hypertrophy [heart weight at harvest and by echocardiography: Left ventricular mass, left ventricular posterior wall thickness at end diastole (LVPWd), and relative left ventricular wall thickness calculated as (LVPWd × 2)/LVIDd]; (b) Measures of heart contractility by echocardiography [left ventricular internal diameters at end diastole (LVIDd), end systole (LVIDs), ejection fraction, fractional shortening]; (c, d) Heart mRNA expression (normalized to sham and housekeeping genes Rpl19 & Hprt) of markers of cardiac overload [atrial natriuretic peptide (ANP), natriuretic peptide B (BNP), and alpha‐Actin 1 (ACTA1)] and fibrosis [TIMP metallopeptidase inhibitor 1 (TIMP‐1), collagen type I alpha 1 (COL1A1), fibronectin 1 (FN1), and matrix metallopeptidase 2 (MMP‐2)]; (e) Representative images of Masson's trichrome‐stained heart tissue sections for fibrosis staining, along with semiquantitative analysis of the percentage of the fibrosis area. Individual values and/or means ± SE are shown. For normally distributed data, one‐way ANOVA and Holm‐Sidak post hoc versus STN‐AngII veh; for non‐normally distributed data, one‐way ANOVA on ranks and Dunn post hoc versus STN‐AngII veh; *n* = 13–19/group for all parameters.

The vehicle‐treated STN‐AngII group had a ~30% greater heart weight versus sham and echocardiography confirmed a similar increase in corrected left ventricular mass, end‐diastolic left ventricular posterior wall thickness, and relative left ventricular wall thickness (Figure 4a). This was associated with a nonstatistical trend for reduced ejection fraction and fractional shortening (Figure 4b), whereas measures of diastolic function were unchanged (Figure S3A). Cardiac gene expression analysis confirmed upregulation of markers of cardiac stress and overload (Nppa, Nppb, Acta1—Figure 4c) and fibrosis (Timp1, Col1a1, Fn1, Mmp2—Figure 4d). The latter was associated with a nonsignificant trend for a greater % area of left ventricular fibrosis staining (Figure 4e).

### 3.3 Evidence for sotagliflozin‐induced target engagement

The mean sotagliflozin dose (calculated based on food intake) was in the targeted range of 10 mg/kg and day (Figure S2C). Consistent with target engagement, sotagliflozin induced a robust glucosuria versus vehicle‐treated STN‐AngII (Figure 2l) despite similar kidney mRNA expression of *Sglt1* or *Sglt2* (Figure S2D). Enhanced glucosuria by sotagliflozin was associated with a nonsignificant trend for lower blood glucose levels (Figure 2m), and a 30% increase in plasma levels of GLP1 versus vehicle‐treated STN‐AngII (Figure 2n). Potentially reflecting body loss of sodium and fluid, sotagliflozin reduced body weight gain when measured after 3 weeks of treatment (Figure 2j), reduced SBP by ~9 mmHg (Figure 2g), and lowered urine creatinine concentrations versus vehicle‐treated STN throughout treatment (Figure S2E). Similar body weight and food intake suggested similar creatinine intake and formation. In this setting, lower urine creatinine concentration can indicate increased urine flow rate. Based on plasma creatinine after 3 weeks of treatment (Figure 2b) and FITC‐sinistrin in the 5th week of treatment (Figure 2c), sotagliflozin temporarily reduced GFR versus vehicle‐treated STN‐AngII by ~40%, while plasma creatinine levels were similarly elevated in both STN‐AngII groups after 6 weeks of treatment at harvest versus sham (Figure 2b).

Potentially related to target engagement (see Discussion for details) sotagliflozin reduced the remaining kidney weight by ~17% versus vehicle‐treated STN‐AngII (Figure 2a). STN‐AngII enhanced the ratio of GFR to kidney mass by ~60% versus the sham group, an effect normalized by sotagliflozin (Figure 2d). Moreover, the small (~4%) increase in heart rate observed in vehicle‐treated STN‐AngII versus sham, was prevented by sotagliflozin (Figure 2h).

### 3.4 Evidence that sotagliflozin did not protect the kidney or heart

Sotagliflozin did not affect the STN‐AngII‐induced rise at harvest in plasma levels of creatinine (Figure 2b) or FGF23 (Figure 2e). Moreover, sotagliflozin did not improve UACR (Figure 2k), or kidney makers of injury, inflammation, and fibrosis versus vehicle‐treated STN‐AngII (Figure 3). Similarly, sotagliflozin did not affect measures of cardiac hypertrophy or molecular markers of cardiac stress, overload and fibrosis in STN‐AngII (Figure 4). Sotagliflozin did not alter measures of cardiac diastolic function (Figure S3A) but modestly reduced cardiac ejection fraction and fractional shortening versus vehicle‐treated STN‐AngII (Figure 4b). This was observed under temporal isoflurane anesthesia, titrated to lower heart rate slightly below 600 beats per minute, which based on experience, induces an anesthesia depth sufficient to perform echocardiography. As a consequence, the ejection fraction was compared under conditions of similar heart rate in all three groups, and heart rates being slightly more suppressed during echo versus awake in vehicle‐treated STN‐AngII than in sham mice (Figure S3B). However, no significant correlation was observed within any of the groups between the heart rate during echocardiography or its change versus awake, respectively, and the ejection fraction (Figure S3C). The data indicate that the small changes in heart rate during echocardiography seem unlikely to explain any observed phenotype in ejection fraction.

## 4 DISCUSSION

In the current study, a murine model of STN was combined with continuous low dose AngII‐infusion. The model showed a 40% reduction in GFR and a ~ 50 mmHg higher SBP compared with sham controls. A doubling in plasma FGF23 levels helped to stabilize plasma phosphate levels. Pressure natriuresis and hormonal counterregulation, indicated by a ~ 50% reduction in kidney mRNA expression of renin, likely helped to maintain sodium and fluid balance and body weight despite maintaining normal food and, thus, sodium intake. The model presented a 100‐fold increase in UACR and robust signs of kidney injury, inflammation, and fibrosis compared with the sham group. This was associated with a ~ 30% larger left ventricle and heavier heart, associated with a similar increase in left ventricular wall thickness and enhanced expression of markers of cardiac volume load and fibrosis. Application of sotagliflozin in the diet established the targeted daily drug dose (~10 mg/kg body wt and day) and engaged its two targets, SGLT2 and SGLT1, respectively. This conclusion is based on the observations that sotagliflozin induced a robust glucosuria, a 9 mmHg reduction in SBP, and a temporal reduction in GFR and body weight. While these changes may be primarily derived from SGLT2 inhibition, a role for tubular SGLT1 inhibition may also have contributed, as discussed below. SGLT1 inhibition was particularly suggested by the observed 30% increase in plasma levels of GLP1 by sotagliflozin versus vehicle. Despite this evidence of target engagement, however, sotagliflozin did not alter at harvest the urinary albumin to creatinine ratio or plasma levels of creatinine or FGF23, kidney markers of injury, inflammation and fibrosis, or cardiac markers of hypertrophy, volume overload or fibrosis.

A few distinct effects of sotagliflozin worth discussing were, nevertheless, observed. The STN‐AngII model showed a ~4% increase in heart rate as measured in awake mice using the automated tail‐cuff system. The STN‐AngII induced NaCl retention and rise in SBP is expected to reduce heart rate as a consequence of baroreceptor activation. For example, using the same automated tail‐cuff system, a salt‐sensitive reduction in heart rate had been observed in mice that, due to a knockout in the P2Y_2_ receptor, present with kidney sodium retention (Pochynyuk et al., 2010; Rieg et al., 2007). In the STN‐AngII model, the expected heart response was potentially more than off‐set by the well‐known inhibitory effect of AngII on the baroreceptor reflex as well as the AngII‐induced stimulation of sympathetic tone and inhibition of parasympathetic tone, both increasing heart rate (Miller & Arnold, 2019; Shanks & Ramchandra, 2021). In this regard, the modest reduction in SBP by sotagliflozin in the STN‐AngII model was associated with a reduced and normalized heart rate. An inhibitory effect of SGLT2 inhibitors on sympathetic tone has been established (Vallon & Verma, 2021; van Ruiten et al., 2022). Whether an inhibition in sympathetic tone could also contribute to the observed reduction in cardiac contractility by sotagliflozin in the STN‐AngII model remains to be determined.

Treatment with sotagliflozin or vehicle was started 1 week after the last surgery with the intention to leave the expected compensatory increase in kidney weight post resection largely unaffected. Using this strategy, sotagliflozin reduced the kidney weight at harvest in the STN‐AngII model. This could be a consequence of the temporal reduction in GFR by sotagliflozin and, thereby, the tubular transport load, which is an important determinant of kidney size and growth. For example, coordinated increases in GFR and kidney size are being observed in pregnancy or a high protein diet (Hussein & Lafayette, 2014; Ko et al., 2020). The GFR‐lowering effect of SGLT2 inhibition has been well established, and is thought to involve enhancing NaCl and fluid delivery to the macula densa thereby activating tubuloglomerular feedback (TGF) as well as a rise in backup pressure in Bowman space (Vallon & Thomson, 2020). A previous study found that the absence of *Sglt1* in mice did not affect GFR and kidney weight in the nondiabetic setting but significantly attenuated the increase in GFR and kidney weight in a genetic model of type 1 diabetes despite no or only minor effects on hyperglycemia (Song et al., 2019). How could SGLT1 inhibition lower GFR/hyperfiltration or kidney growth/weight? When the level of filtered glucose overwhelms SGLT2‐mediated glucose uptake or SGLT2 is inhibited, large amounts of glucose can be reabsorbed through downstream SGLT1 in late proximal tubule and thick ascending limb (Powell, DaCosta, et al., 2013; Rieg et al., 2014; Song et al., 2019). Inhibition at these sites of SGLT1, which transports 2 Na^+^ per glucose (while SGLT2 transports only 1 Na^+^ per glucose), could reduce GFR through TGF and an increase in tubular backup pressure, as described for SGLT2i (Vallon & Thomson, 2020). In addition, SGLT1 via its expression in the luminal membrane of the macula densa serves as a glucose sensor that is linked to local nitric oxide formation, thereby contributing to diabetic hyperfiltration (Song et al., 2019; Zhang et al., 2019, 2022) and potentially diabetic kidney growth (Song et al., 2019). The hypertensive STN model itself did not induce glucosuria, arguing that glucose delivery to the macula densa was likely not robustly increased. The SGLT2i component of sotagliflozin, however, delivers more glucose to macula densa SGLT1 and could increase macula densa NOS1 expression, as previously shown for the selective SGLT2i, dapagliflozin, in nondiabetic mice (Song et al., 2019). This could attenuate the GFR‐lowering effect of selective SGLT2i that is caused by increased macula densa NaCl and fluid delivery. Additive GFR‐lowering effects of an SGLT2 inhibitor and genetic knockdown of *Sglt1* have been shown in hyperfiltering diabetic mice (Song et al., 2019). Thus, sotagliflozin could have a stronger GFR‐lowering effect than a selective SGLT2i. However, it is currently unknown to which extent sotagliflozin reaches and inhibits tubular SGLT1, including in the late proximal tubule and macula densa. In this regard, absence of *Sglt1* in mice improved the kidney recovery in a nondiabetic model of kidney ischemia reperfusion (Nespoux et al., 2019), but comparative studies with sotagliflozin have not been performed. Moreover, the implications of sotagliflozin normalizing the GFR to kidney weight ratio in response to STN‐AngII remains unclear.

As outlined in the Introduction, SGLT2 inhibitors have been shown to be kidney protective in individuals with and without T2DM, and sotagliflozin in individuals with T2DM. While the current study has multiple strengths, like (1) combination of a CKD model with hypertension, (2) assessment of kidney and cardiac function, and (3) combining physiological, metabolic and biochemical assessments, the study also has limitations that may explain why broader cardio‐renal protection was not observed with sotagliflozin. There is some evidence in humans that the kidney protection of SGLT2 inhibitors is somewhat stronger in the presence of T2DM (Heerspink et al., 2021) and the mice in the current study were not diabetic. It is also possible that study treatment was too short or the model not suitable to capture benefits of the initial GFR lowering. In this regard, the current model with its severe hypertension and unopposed AngII tone may show little responsiveness to kidney protective strategies other than AngII blockade, and could therefore be of limited value for the exploration of alternative strategies. The SGLT2 inhibition‐induced transport shift and hyperreabsorption in tubular segments downstream of the early proximal tubule may be magnified in the STN‐AngII model and promote medullary hypoxia, particularly if sotagliflozin did not effectively inhibit tubular SGLT1. SGLT2 inhibitors were previously reported to improve kidney outcome in multiple rodent models of nondiabetic 5/6 nephrectomy (Chen et al., 2023; Matsui et al., 2024; van der Pluijm et al., 2024; Yang et al., 2024) but not in all instances (Zhang et al., 2016), and the STN‐AngII model had not previously been tested in this regard. Notably, SGLT2 inhibition was likewise not kidney protective when heminephrectomy was combined with tonic mineralocorticoid receptor activation and high salt treatment (Tauber et al., 2021).

In summary, in a nondiabetic murine model of subtotal nephrectomy with low dose AngII‐driven hypertension, the initiation of the dual SGLT2/SGLT1 antagonist, sotagliflozin, one week after the last surgery, induced evidence for target engagement, including glucosuria, modest reduction in systolic blood pressure, temporal reduction in GFR, and an increase in GLP1 plasma levels, but did not improve signs of kidney injury, inflammation, fibrosis and albuminuria or signs of cardiac overload, fibrosis, or dysfunction.

## FUNDING INFORMATION

The authors were supported by National Institutes of Health (NIH) Grants R01 DK112042 and P54 DK137307 (University of Alabama at Birmingham/University of California‐San Diego O'Brien Center of Acute Kidney Injury Research), the Department of Veterans Affairs, and an investigator‐initiated research project funded by Lexicon Pharmaceuticals (all to V. V.).

## CONFLICT OF INTEREST STATEMENT

Over the past 24 months, V. Vallon has served as a speaker/consultant and received honoraria from Astra‐Zeneca and Boehringer Ingelheim and received grant support for investigator‐initiated research from Boehringer Ingelheim, Gilead, Lexicon, Maze Therapeutics, and Novo‐Nordisk. V. Vallon had full control over the design of the study, the decision to publish, and the contents of the manuscript. None of the other authors has any conflicts of interest, financial or otherwise, to disclose.

## ETHICS STATEMENT

All animal experimentation was conducted in accordance with the Guide for Care and Use of Laboratory Animals (National Institutes of Health, Bethesda, MD) and was approved by the local Institutional Animal Care and Use Committee of the Veterans Affairs San Diego Healthcare System.

## Supporting information


Figures S1–S3.

## Data Availability

All data generated or analyzed during this study are included in this article. Further enquiries can be directed to the corresponding author.
