# Supplementary figures and images for: Effects of sotagliflozin on kidney and cardiac outcome in a hypertensive model of subtotal nephrectomy in male mice
Source: Physiol Rep. 2025 Mar 28;13(7):e70217. doi: 10.14814/phy2.70217 (PMC11950634; doi:10.14814/phy2.70217)

Fig. S1

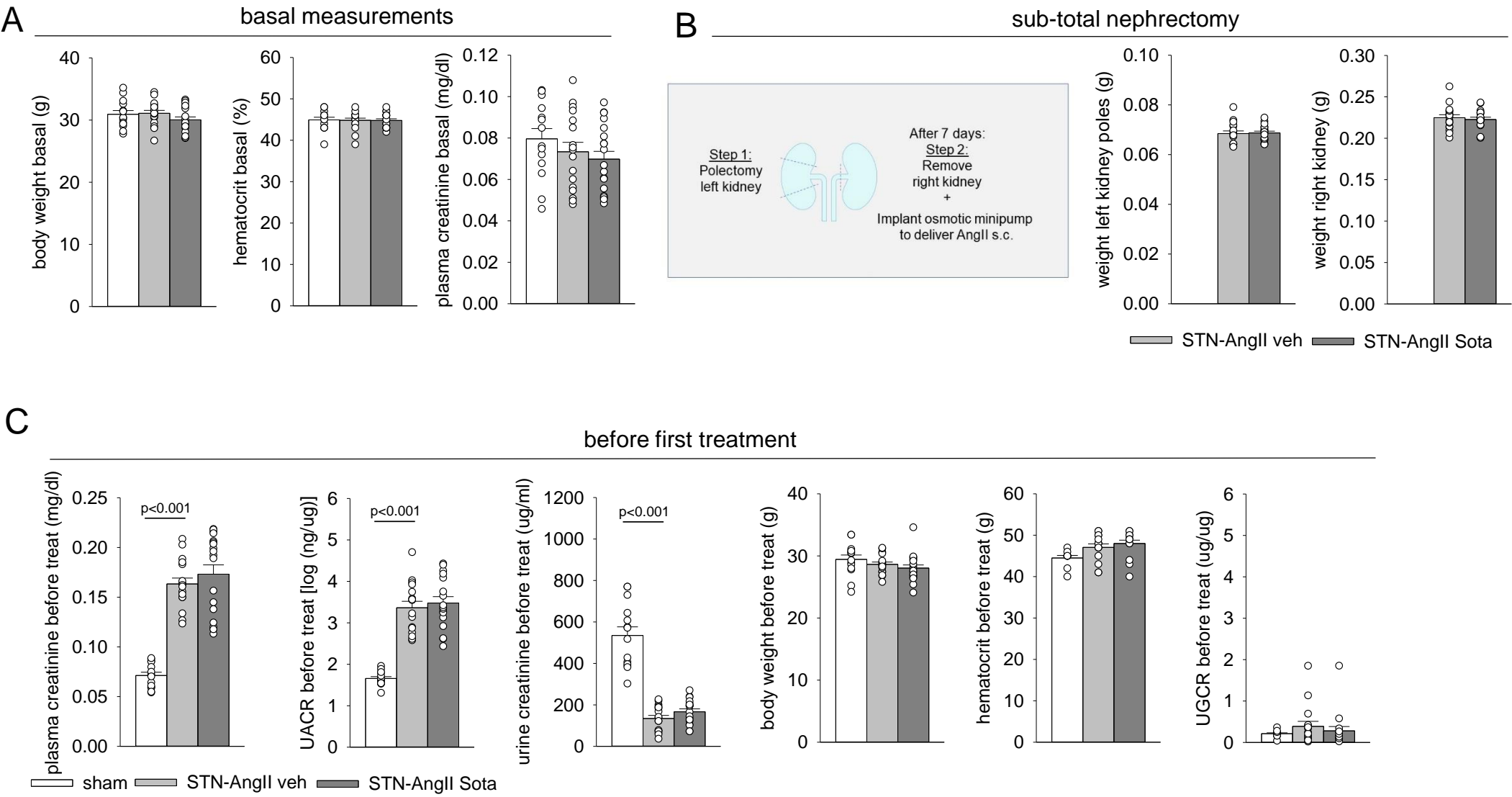

Fig. S2

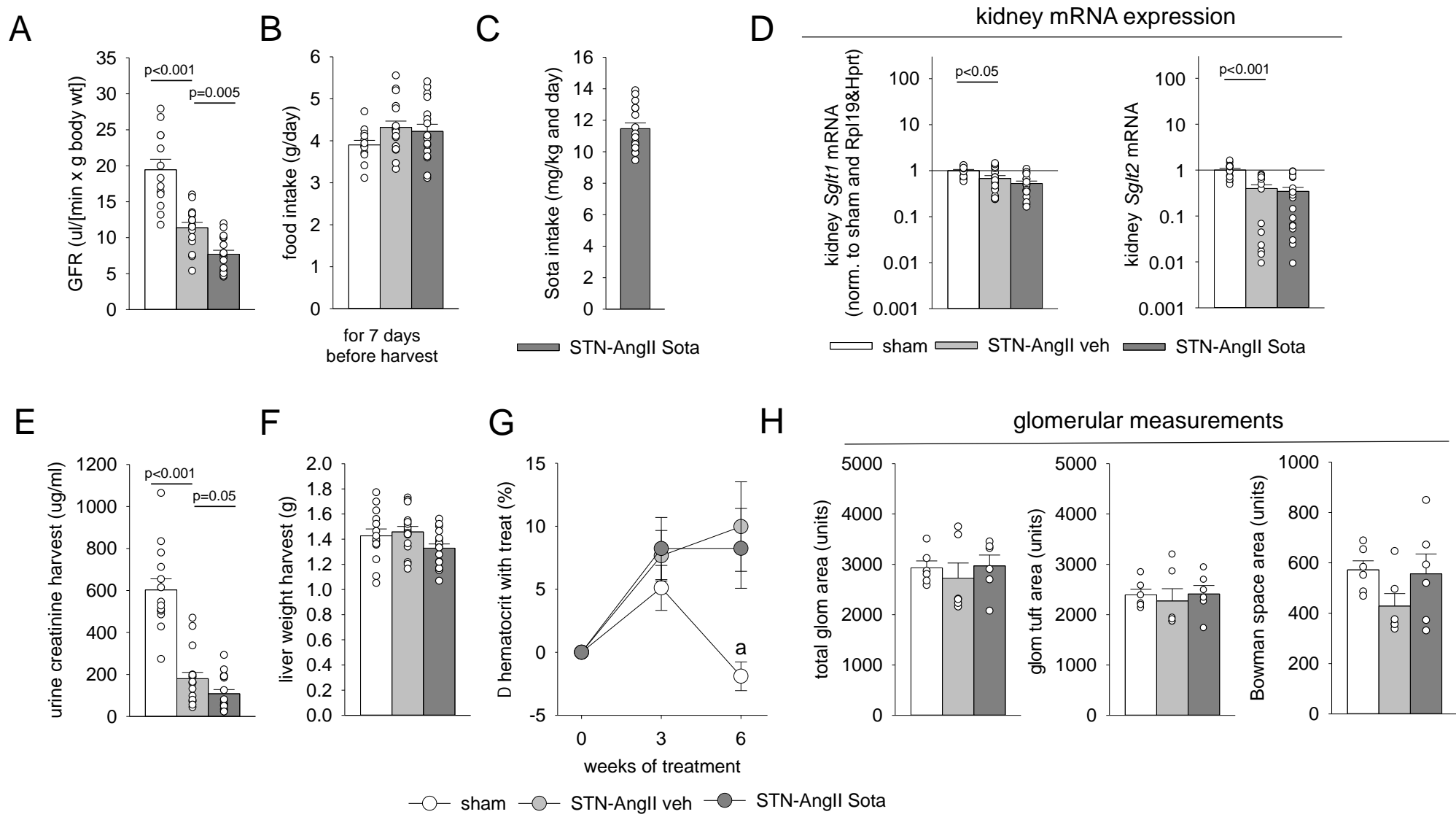

Fig. S3

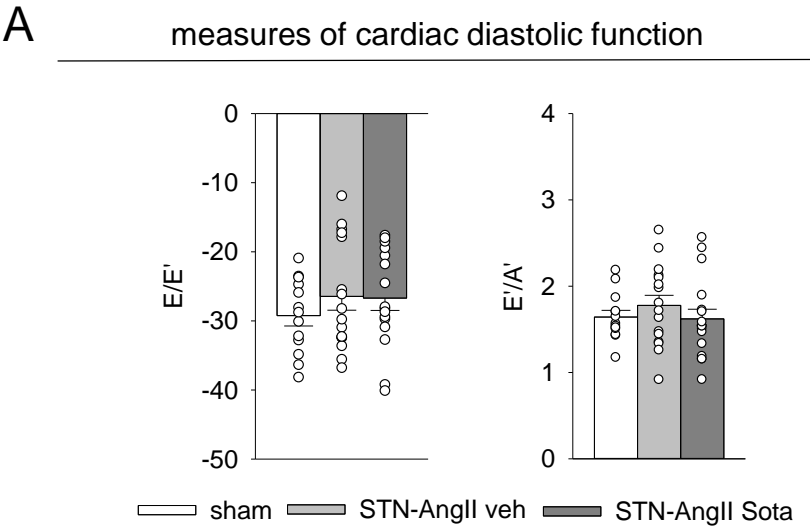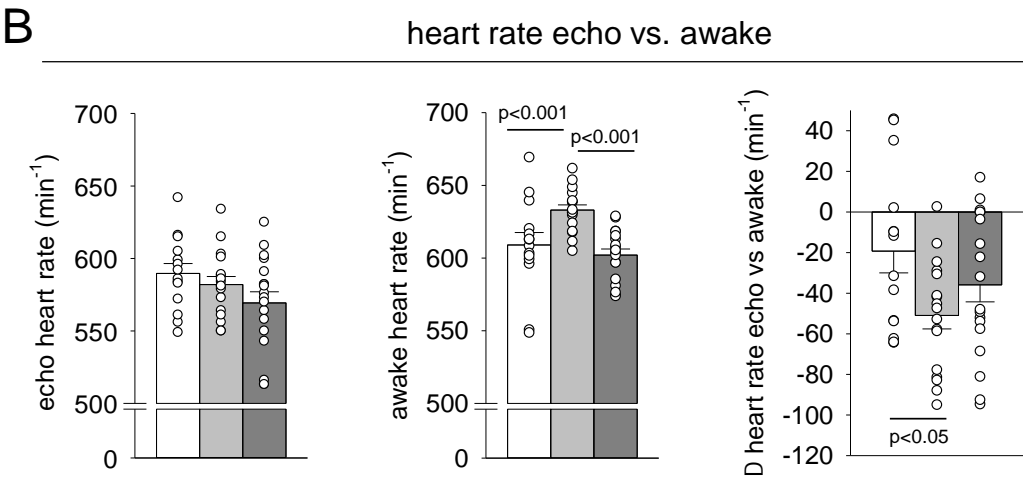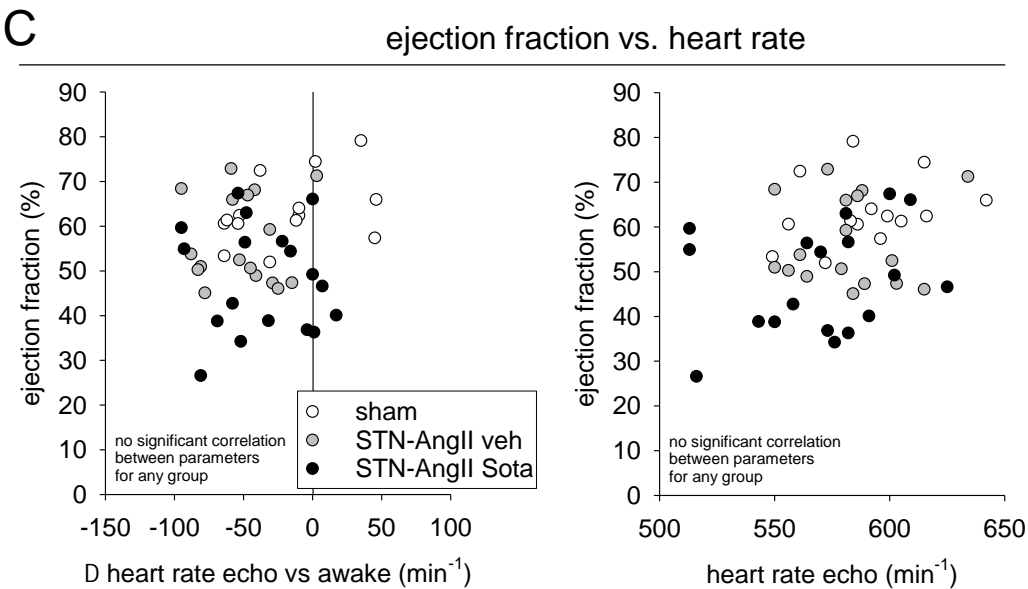

Supplement: Supplementary file 1 — Figures S1–S3. [file PHY2-13-e70217-s001.pdf]
